# Sleep better, feel better? Effects of a CBT-I and HT-I sleep training on mental health, quality of life and stress coping in university students: a randomized pilot controlled trial

**DOI:** 10.1186/s12888-018-1860-2

**Published:** 2018-08-29

**Authors:** Anja Friedrich, Merle Claßen, Angelika A. Schlarb

**Affiliations:** Faculty of Psychology and Sports Science, Department of Psychology, Clinical Psychology and Psychotherapy of children and adolescents, Bielefeld University, Universitätsstraße 25, 33615 Bielefeld, Germany

**Keywords:** Sleep, Mental health, Quality of life, Stress coping strategies, CBT-I, HT-I

## Abstract

**Background:**

The SWIS sleep training for university students showed promising results regarding subjective and objective sleep parameters. As sleep disorders and impaired sleep quality are closely related to various aspects of mental health, the current study examines the effects of the SWIS sleep training on mental health in university students.

**Methods:**

Fifty six university students (M = 25.84, SD = 5.06) participated in the study, 68% were women. Forty one were randomly assigned to the SWIS treatment (pre-post-follow-up), 15 to a Waiting List Control condition (WLC, pre-post). Besides sleep-related measures, the students completed four online questionnaires measuring mental health, quality of life and stress coping strategies. Effect sizes for the pre-post data were compared between the conditions, long-term effects were calculated with repeated measures ANOVA or Friedman ANOVA. Long-term clinical changes were analyzed with the Reliable Change Index (RCI).

**Results:**

The pre-post comparisons between SWIS and WLC revealed lower depression scores in both conditions, a better physical state in the SWIS condition and less maladaptive stress coping strategies in the WLC students. The long-term results of SWIS provided significant improvements regarding the students’ somatic complaints, reduced anxiety, an improved physical state and a better quality of life with moderate to large effect sizes. Most of the significant improvements occurred between pre- and follow-up measurement. These statistically significant results were also reflected in clinically significant changes from pre- to follow-up-test.

**Conclusions:**

SWIS and WLC condition both improved in two mental health variables immediately after the training. These findings may be explained by unspecific treatment expectation effects in the WLC. Interestingly, most mental health outcomes showed significant improvements after 3 months, but not immediately after the training. These positive long-term effects of the SWIS training on mental health indicate that the transfer of strategies might simply need more time to affect the students’ mental health.

**Trial registration:**

The current study was retrospectively registered at German Clinical Trials Register (ID: DRKS00014338, registration date: 20.04.2018, enrolment of first participant: 14.04.2015).

## Background

### Sleep in college and university students

Sleep problems are quite common among university students: Up to 16% need more than 30 min to fall asleep and 7.7% meet the diagnostic criteria for an insomnia disorder [1]. Aside from insomnia symptoms, more than one third (36.9%) of German college students report a poor sleep quality according to the Pittsburgh Sleep Quality Index [2]. Other studies found even higher percentages of impaired sleep quality (42.8%) in German and Luxembourgian university students [3].

### Sleep and mental health

Sleep and sleep problems are closely related to various mental health variables in university students. In a sample of 1074 American college students (72% women), those with chronic insomnia according to DSM-5 (9.5%) reported significantly more fatigue, depression, anxiety, stress and stimulant use than healthy students [4]. In another study, Lund and colleagues examined the differences between good and bad sleepers according to the Pittsburgh Sleep Quality Index [5]. Of the 1125 American participants (63% women), two thirds were classified as bad sleepers. They reported delayed bed- and risetimes during the weekend, more anger, confusion, depression, fatigue and tension, more physical illness, daytime sleepiness as well as more drug and alcohol consumption [5]. In addition, Lund and colleagues showed that especially academic and emotional stress explained the students’ sleep quality, while alcohol, caffeine consumption and sleep consistency were of lesser importance [5]. A recent study from Ethiopia examined 2654 college students (24% women) and found that general health (e.g. sadness, mastering daily problems, self-esteem) is significantly correlated with sleep quality and daytime sleepiness [6]. Furthermore, these correlations continued to exist even when controlling for demographic and behavioral covariates. Similar results were observed for German college student samples with impaired sleep quality that reported more mental strain, somatic complaints, as well as lower self-efficacy [2, 3]. Overall, current research shows that not only sleep disorders, but also impaired sleep is connected with mental health in university students.

### Sleep and stress coping strategies

While the associations between sleep and mental health have been investigated extensively, the role of stress coping behaviors in college students remains vague. In a sample of 2892 American college students (59% women), 9.1% suffered from insomnia according to DSM-IV [7]. In this study, stress exposure was a significant predictor of insomnia onset and the relationship was mediated by the three stress coping strategies behavioral disengagement, distraction and substance abuse [7].

### Sleep and quality of life

Besides mental health and stress coping strategies, the quality of life is another factor that is closely related to the students’ sleep. Students with chronic insomnia reported a significantly lower quality of life, including worse physical health, more negative emotions, less leisure time activities, household duties and course work [4, 8]. Preišegolavičiūtė and colleagues examined a broader concept that included the students’ work and studies, emotional status, physical health and sexual life [9]. The analysis of 405 Lithuanian students (73% women) showed that impaired sleep quality was significantly correlated with a reduced quality of life.

### Sleep interventions

All in all, the current research shows that sleep in university students is associated with mental health problems, stress coping strategies and quality of life. Correspondingly, an improvement of the students’ sleep quality should also improve their mental health, stress coping and quality of life. This assumption was partially confirmed in a recent review about different psychological interventions to improve sleep in university students [10]. Regarding the improvements of the students’ mental health, sleep hygiene provided small effects, whereas cognitive behavior therapy (CBT) and other approaches showed medium effects, and relaxation trainings had large effects [10]. Hence, it is possible to enhance university students’ mental health via sleep trainings. However, the students’ stress coping and quality of life were not addressed in this review, as they were only reported in a few studies.

### “Studieren wie im Schlaf”

An example of the aforementioned sleep trainings is “Studieren wie im Schlaf” (SWIS). SWIS is a group training designed to improve sleep in college and university students. It combines cognitive behavior therapy for insomnia (CBT-I) and hypnotherapy for insomnia (HT-I). With only six sessions à 100 min, SWIS is a short but effective training. In a pilot study, SWIS was a well-accepted and feasible program that improved subjective as well as objective sleep impairments [11].

Furthermore, similar sleep trainings that consist of CBT-I and HT-I proved effective in infants, children and adolescents (*infants* [12–14]; *children* [15, 16]; *adolescents* [17, 18]). These studies with younger children and adolescents showed that the improvement were largest after 3 months [16]. This incubation effect might be caused by two factors: the parents need time to transfer changed parental sleep-related behavior into daily routine, furthermore, children need to adapt newly learned self-regulated sleep strategies into autonomous behavior.

### Objectives and hypotheses

As described above, sleep is closely related to a plethora of mental health aspects. Therefore, we assumed that besides the improvement of sleep, the SWIS training may result in i) a greater reduction of the students’ mental health problems than the waiting list control condition (WLC) including less depression, somatic complaints, panic, anxiety, alcohol abuse, and general stress. Beyond, we hypothetized that ii) the SWIS training improves the students’ stress coping strategies with a greater increase in the use of adaptive strategies and a greater reduction of maladaptive strategies in comparison to the WLC. In addition, that iii) students of the SWIS training report greater improvement of quality of life than students of the WLC. Finally and most important, iv) that the effects of the SWIS training are sustained or improved in the long-term. These long-term effects are greater for students with clinically relevant depressive symptoms.

## Method

### Intervention: Studieren wie im Schlaf (SWIS)

SWIS is conducted in groups with four to eight students. In six manualized weekly sessions, the students learn different strategies to cope with and improve their sleep problems and sleep behavior. These strategies include CBT-I and HT-I. The behavior therapeutic elements encompass sleep hygiene, progressive muscle relaxation, cognitive restructuring, stress management and problem solving skills. The hypnotherapeutic elements include sleep related trances, calming pictures, self-hypnosis and imagination techniques. Each session (except for the first) starts with each participants’ report on their individual progress and the daily implementation of the sleep-related strategies. Afterwards follows the manualized content. Every session concludes with a trance session. A more detailed description is provided in a recent article by Schlarb, Friedrich and Claßen [11].

### Procedure

**Fig. 1:**
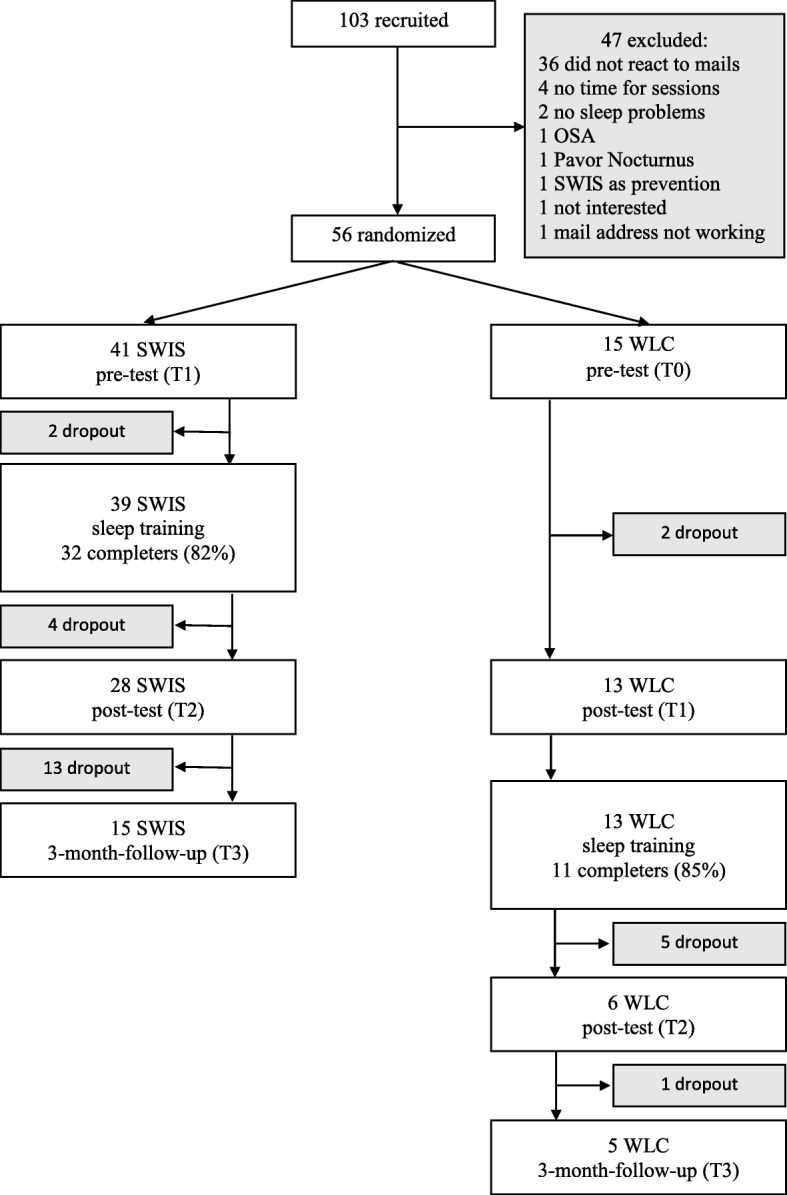
Flow chart of the experimental design. One hundred three participants were recruited, 56 were randomized (SWIS: *N* = 41, WLC: *N* = 15). After the waiting period, the WLC received the SWIS sleep training. Completion rates were 82% in the SWIS condition and 85% in the WLC condition. All participants who completed at least four of the six sessions were counted as completers. Dropout rates aside from non-completers were 46% in the SWIS condition and 53% in the WLC condition

An experimental pre-post-follow-up design was employed. Students were randomly assigned to the SWIS intervention condition (SWIS) or a waiting-list control condition (WLC) in a 3:1 ratio (Fig. 1). All in all, 56 participants were included. All participants provided written informed consent. The SWIS training was guided by two psychologists who were supervised by a psychotherapist. The study pertains to the Declaration of Helsinki and was approved by the ethics committee at Bielefeld University (ID: 2014–135).

The participants of SWIS completed the pre-test (T1) including a self-developed diagnostic interview to check inclusion and exclusion criteria and a 2 week sleep log. Afterwards, the SWIS training was conducted, which lasted 6 weeks, immediately followed by the post-test (T2). The follow-up test was administered after 3 months (T3). The participants in the WLC first received a 2 week pre-test (T0) and then waited for 6 weeks, after which they completed the post-test (T1). Due to ethical reasons, they received the same procedure as the intervention condition. If WLC and SWIS did not differ at T1, the WLC data were included in the long-term analyses.

### Inclusion and exclusion criteria

For inclusion in the study, participants had to fulfill two criteria. First, they had to report symptoms of an insomnia disorder, a nightmare disorder or an irregular sleep-wake type according to DSM-5 criteria [19]. Secondly, based on the Pittsburgh Sleep Quality Index [20], their sleep quality was classified as poor (sum score > 5).

The inclusion and exclusion criteria were checked during the self-developed diagnostic interview. Participants with untreated organic diseases were excluded from study participation. Participants with treated organic diseases (mostly thyroid disorders) were included in the study, provided that they received continuous adequate medical treatment and that the organic disease was not the main cause of the sleep problems. Consequently, participants with reported symptoms of organic sleep disorders (such as obstructive sleep apnea) were excluded from the study. If participants presented symptoms of organic sleep disorders but were not yet diagnosed, they were referred to a sleep laboratory for further diagnosis.

Participants with previously treated psychiatric disorders were included in the current study, provided that the psychiatric disorder was not the main cause of the sleep problems and that they did not receive psychotherapy simultaneously. An exemption to this rule were participants with current psychotic episodes, trauma-related disorders, or cognitive impairments, which were excluded in general.

### Sample

The sample size was computed with G*Power, version 3.1.9.2 [21]. With an alpha error of .05, a power of .95 and an estimated effect size for CBT sleep trainings in university students of d = .59 [10], the sample size had to exceed 33 participants for dependent t-tests.

The participants were recruited at Bielefeld University using leaflets, Email and a homepage. The sample consisted of 56 university students aged 19 to 50 years (M = 25.84, SD = 5.06) (Table 1). Sixty eight percent of the participants were women. Forty one were assigned to SWIS, 15 to the WLC. A Chi-square test and a Wilcoxon signed rank test were used to investigate group differences between SWIS and the WLC. Both tests provided statistical evidence that the groups did not differ regarding gender or age. One student did not complete the online questionnaires and was therefore excluded from the following analysis.

**Table 1:** Sample characteristics of SWIS and WLC

N	all	SWIS	WLC	SWIS vs. WLC
	56	41	15	
Gender				X^2^_gender_(*N* = 56) = 1.981, *p* = .202
women	38 (68%)	30 (73%)	8 (64%)	
men	18 (32%)	11 (27%)	7 (36%)	
Age				Z_age_ = −1.471, *p* = .141
mean	25.84	25.13	27.73	
SD	5.06	3.82	7.26	

A more detailed analysis of the age range revealed that 87.3% of the sample were younger than 30 years of age. Thus, only 12.7% were older than the “usual” student sample. To ensure that the older students do not differ from the younger students, baseline differences in all dependent variables between younger (19–30, *N* = 49) and older students (31–50, *N* = 7) were examined using Mann-Whitney-U tests for independent nonparametric samples. These analyses revealed that there were no differences between the younger and the older students (all *p*-values > .10).

### Instruments

#### Depression

Based on the Center for Epidemiological Studies Depression Scale (CES-D), Hautzinger and Bailer generated the German *General Depression Scale* (Allgemeine Depressionsskala, ADS) [22]. It is composed of 20 items that gauge the subjects’ moodiness and tendency towards depressive symptoms during the last week on a four point rating scale ranging from zero (rarely) to three (mostly). Higher values indicate a higher tendency towards depressive symptoms. The ADS showed good internal consistency (α = .89) and a reliable cut-off score of 23 [22]. The cut-off score was used to divide the participants into participants with clinically relevant depressive symptoms (ADS ≥23) and participants without clinically relevant depressive symptoms (ADS < 23) for the long-term analyses.

#### Somatoform complaints, panic, anxiety, alcohol abuse and general stressors

Four scales from the German version of the *Patient Health Questionnaire* (PHQ-D [23]) were used to screen for the following mental health issues: Somatic complaints (13 items, 0–26), panic (11 items, 0–11), anxiety (6 items, 0–12), alcohol abuse (5 items, 0–5) and general stressors (9 items, 0–27). The three scales panic, anxiety and alcohol abuse were only presented after a filter question was confirmed (e.g. “Do you sometimes consume alcohol (including beer and wine?”)). Therefore, not all participants completed these three scales. Gräfe, Zipfel, Herzog and Löwe analyzed the psychometric qualities and confirmed construct validity, specificity, sensitivity, patient and physician acceptance as well as internal consistency (α = .79–.88) [24]. However, the authors only reported the internal consistency of some subscales (e.g. somatic complaints), as they used several subscales categorically (e.g. anxiety). Therefore, the reliabilities for the other subscales will have to be computed in this study. The test retest reliability was not provided for the subscales used in this study.

#### Stress

The short form of the *Stress Coping Questionnaire* (Stressverarbeitungsfragebogen, SVF-78 [25]) consists of 78 items that assess 20 different coping strategies for stressful situations, for example avoidance, aggression and relaxation. Two secondary scales, adaptive and maladaptive strategies, summarize ten and seven subscales respectively. Higher values indicate a higher probability for the application of a coping strategy. The SVF-78 shows internal consistency (α = .77–.94) and construct validity [25]. However, test retest reliability is only available for children and adolescents, not for adults.

#### Quality of life

The *Quality of Life Scales* (Skalen zur Erfassung der Lebensqualität, SEL [26]) contain 28 items and six scales about the subject’s current mood, objective physical complaints, subjective physical condition, predominant mood, environment and general attitude towards life. These scales are combined into two global scales: Physical condition and cognitive-emotional condition. These two global scales add up to the sum score “life quality”. Two global items concerning body (“How do you rate your physical condition in general?”) and quality of life (“In general, how do you rate your life quality?”) are added.

Participants respond on three different five point Likert scales, ranging from “never/does not apply to me at all/bad” (1) to “always/applies to me completely/very good” (5). Higher scores indicate a higher quality of life. In this study, only the two global scales physical condition, cognitive-emotional condition and the sum score were analyzed.

Although Averbeck and colleagues provide norms for healthy adults (*N* = 1683), they were not used for data analysis due to the different population characteristics. The global scales physical condition (α = .88) and cognitive emotional condition (α = .92) and the quality of life scale (α = .94) showed excellent reliabilities [26]. Test retest reliabilities were only tested in tumour patients; hence they are not applicable to the current target population. Scale intercorrelations range from .27 to .62 [26].

### Data analysis

All calculations were computed with the Statistical Package for the Social Sciences version 22 [27]. First, all variables were examined for normal distribution (Shapiro-Wilk). Then, they were tested for baseline differences between SWIS and WLC with a t-test for independent samples (normally distributed), a Mann-Whitney-U test (not normally distributed), or a Chi-square test for nominal variables (depression). The missing reliability coefficients of the PHQ-D subscales anxiety and general stress were computed using Cronbach’s alpha.

#### Pre-post comparisons between SWIS and WLC

The differences between the pre- and post-tests were tested for normal distribution (e.g. ADS_pre_ - ADS_post_ = ADS_diff_). As all differences were normally distributed, only parametric tests were computed. A repeated measures ANOVA would have been the standard test for the first three hypotheses. However, the small and unequal sample sizes did not provide sufficient statistical background for an ANOVA. Instead, the two conditions (SWIS vs. WLC) were analyzed separately with dependent t-tests (pre vs. post). Then, effect sizes were calculated and compared descriptively.

#### Long-term effects of SWIS

For the fourth hypothesis, the pre-, post- and follow-up data were tested for normal distribution. According to normal distribution, the long-term effects of the SWIS training were examined with ANOVA or Friedman’s ANOVA. The post-hoc tests were adjusted for alpha error accumulation. In this case, the ANOVA was the most feasible approach, as only one condition was examined over the course of time. If the SWIS and the WLC condition did not differ at T1, the WLC data were included in the long-term analyses.

In order to test the effect of depressive symptoms on the long-term development of the students’ mental health, a between subjects factor was added that sorted the participants into two groups according to the ADS (with or without clinically relevant depressive symptoms).

#### Long-term clinically significant changes

Long-term clinically significant changes were examined by calculating the Reliable Change Index (RCI) for each instrument. These scores were then applied to the instruments’ change scores that were calculated using the formula:

$$ {\mathrm{X}}_{\mathrm{change}}={\mathrm{X}}_{\mathrm{pre}}-{\mathrm{X}}_{\mathrm{follow}\hbox{-} \mathrm{up}} $$

The RCIs were then applied to the instruments’ pre- to follow-up change scores, classifying them into deterioration, no change and improvement. In the current study, the calculation of the RCIs followed Jacobson and Truax [28] with a 5% alpha error:

1. Calculate the instruments’ pre-test standard error, S_E_ = s_pre_√(1-r_xx_).
  s_pre_ as the standard deviation of the instruments’ pre-test score.
  r_xx_ as the instruments’ internal consistency.
2. Calculate the instruments’ standard deviation of the errors, S_Diff_ = √(2(S_E_)^2^).
3. Calculate the RCI = S_Diff_ * 1.96.

Example depression (ADS):

Calculate the instruments’ pre-test standard error, S_E_ = 9.71√(1–.89) ≈ 3.22.
2) Calculate the instruments’ standard deviation of the errors, S_Diff_ = √(2(3.22)^2^) ≈ 4.55.
Calculate the RCI = 4.55 * 1.96 ≈ 8.92 ≈ 9.

Interpretation:

The RCI would be 9 points on the ADS. As the change scores are computed ADS_pre_ – ADS_follow-up_ and higher scores indicate more depressive symptoms, a change score of + 9 points would indicate a clinically significant improvement (as the pre-test score would be reduced by 9 points) while a change score of − 9 points would indicate a clinically significant deterioration (as the pre-test score would increase by 9 points). Change scores that lie between − 9 and + 9 would indicate no clinical change.

#### Completer analyses

Completer analyses were conducted to test whether post- (T2) and follow-up (T3) completers differed from non-completers at T1 (post-completers vs. non-completers) or T2 (follow-up completers vs. non-completers). Participants were compared using independent t-tests (normally distributed) or Mann-Whitney-U tests (not normally distributed). Outcome measures were the same mental health, stress coping and life quality variables as in the pre-post comparisons.

#### Missing data and effect sizes

Due to the small sample size and the concurrent higher variance, missing data were not imputed but excluded pairwise. The alpha level was set to *p* < .05, a tendency was detected at *p* < .10. There was no adjustment for alpha error accumulation because of the small sample size and the explorative character of the study, except for the post-hoc tests for the last hypothesis. Effect sizes were calculated for all significant results and tendencies. Cohen’s d^1^ was used for the parametric tests, with small (d > .20), medium (d > 50) and large effects (d > .80). For nonparametric tests, r [29]^2^ was also categorized into small (*r* > .10), medium (*r* > .30) and large effects (*r* > .50).

## Results

### Baseline characteristics and differences between SWIS and WLC

Table 2 displays the baseline means and standard deviations for SWIS and WLC. The reliabilities of the PHQ-D subscales anxiety (α = .69) and general stress (α = .70) were deemed acceptable. The scales somatic complaints, alcohol abuse, adaptive stress coping strategies and physical condition were not normally distributed and analyzed accordingly. As Table 2 demonstrates, there were no statistically significant differences between SWIS and WLC at baseline. On average, all students in both groups reported high depression scores, as 56% of the students exceeded the ADS cut-off score. 54% of the SWIS condition and 62% of the WLC condition showed clinically relevant depressive symptoms. Panic symptoms and alcohol abuse were uncommon (N_panic_ > 0 = 1; N_alcohol_ > 0 = 5). Therefore, these two scales were excluded from further analysis.

**Table 2:** Baseline differences between SWIS and WLC

Variable	SWIS		WLC		Test statistic	p
	N	M / % (SD)	N	M / % (SD)		
Mental health (ADS, PHQ)						
Depression (ADS) 0–60	39	23.54 (8.84)	15	25.40 (7.51)	t(52) = .721	.474
Clinically relevant depressive symptoms (ADS ≥23)	39	21 (54%)	13	8 (62%)	X^2^(52) = .234	.629
Somatic complaints (PHQ) 0–26	39	7.62 (4.71)	15	7.20 (4.59)	Z = −.252	.801
Panic (PHQ) 0–11	11	3.91 (3.14)	6	5.13 (2.36)	t(17) = .919	.371
Anxiety (PHQ) 0–12	38	7.58 (2.68)	14	7.57 (2.56)	t(50) = −.009	.993
Alcohol abuse (PHQ) 0–5	29	.24 (.58)	12	0 (0)	Z = −1.513	.403
General stressors (PHQ) 0–27	39	7.74 (3.40)	15	6.47 (2.89)	t(52) = − 1.284	.205
Stress coping strategies (SVF)						
Adaptive strategies	39	17.97 (2.64)	15	17.28 (3.92)	Z = −.464	.643
Maladaptive strategies	39	19.52 (3.30)	15	19.97 (4.13)	t(52) = .415	.680
Quality of life (SEL)						
Physical condition	39	2.96 (.63)	15	2.72 (.84)	Z = −1.393	.164
Cognitive emotional condition	39	2.79 (.56)	15	2.87 (.62)	t(52) = .440	.662
Quality of life	39	3.13 (.54)	15	3.03 (.50)	t(52) = −.642	.524

N varies across variables due to the fact that not all participants affirmed the filter questions for the three PHQ-scales panic, anxiety and alcohol abuse

### Pre-post comparisons between SWIS and WLC

The comparisons between pre- and post-test are presented in Table 3. Students of both conditions reported significantly less depression at post-test with small (SWIS) and medium (WLC) effects. The SWIS condition additionally showed a significantly better physical condition after the training with a small effect size. WLC students reported significantly less maladaptive stress coping strategies with a small effect size. Furthermore, the participants of the SWIS condition had descriptively less anxiety and general stress after the training, while WLC students reported more anxiety and general stress.

**Table 3:** Separate pre-post comparisons with effect sizes for the two conditions

Variable	N	SWIS		t	p	d	N	WLC		t	p	d
		Pre M (SD)	Post M (SD)					Pre M (SD)	Post M (SD)			
Mental health												
Depression	28	23.43 (8.74)	20.61 (8.05)	**2.367**	**.025**	**.32**	13	24.92 (7.54)	21.46 (6.94)	**2.843**	**.015**	**.56**
Somatic complaints	28	7.75 (4.49)	7.04 (3.84)	1.285	.210		13	6.15 (3.87)	5.69 (2.02)	.496	.629	
Anxiety	28	7.96 (2.47)	7.62 (2.38)	1.104	.280		11	7.00 (2.53)	7.45 (1.97)	−.833	.424	
General stress	28	7.75 (3.05)	7.50 (3.67)	.500	.621		13	6.46 (3.13)	7.23 (4.79)	−.784	.448	
Stress coping strategies												
Adaptive strategies	27	12.32 (2.20)	12.13 (2.70)	.393	.679		13	16.91 (4.08)	16.73 (4.23)	.370	.718	
Maladaptive strategies	27	13.50 (3.47)	12.79 (3.51)	1.522	.140		13	19.23 (3.94)	16.69 (4.52)	**2.449**	**.031**	**.48**
Quality of life												
Physical condition	28	2.95 (.58)	3.27 (.57)	**−2.848**	**.008**	**.38**	13	2.78 (.89)	2.98 (.66)	−1.099	.294	
Cognitive emotional condition	28	2.82 (.58)	2.94 (.56)	−1.246	.223		13	2.89 (.66)	2.98 (.59)	−.574	.577	
Quality of life	28	3.15 (.56)	3.29 (.55)	−1.487	.149		13	3.04 (.53)	3.18 (.56)	−1.067	.307	

Bold characters indicate significant results (*p* < .05)

#### Clinically relevant depressive symptoms

**Fig. 2:**
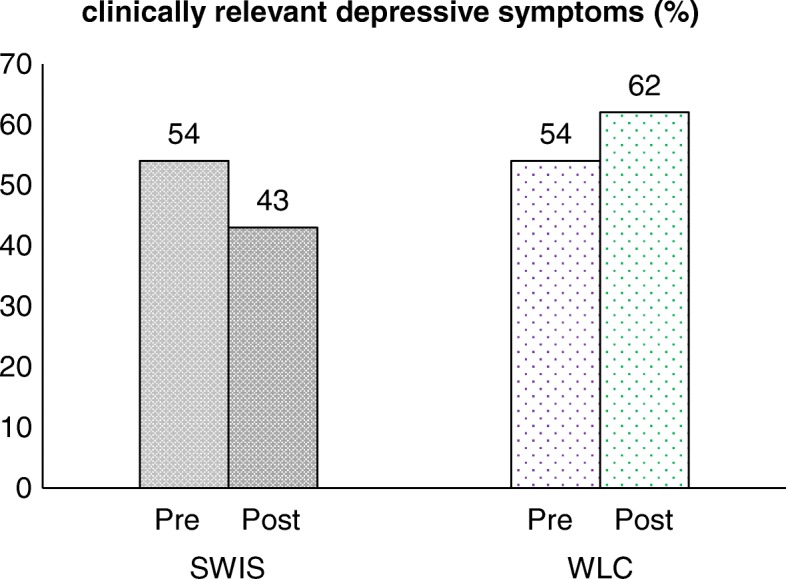
Percentage of participants with clinically relevant depressive symptoms for SWIS (*N* = 28) and WLC (*N* = 13) condition during pre- and post-test. The pre-test percentages may vary from Table 2 as only participants who completed both measurements were included

Figure 2 displays the percentage of the participants that report clinically relevant depressive symptoms (ADS ≥23) for each condition during pre- and post-test. The percentages of participants with clinically relevant depressive symptoms did not differ between the two conditions at pre-test. However, fewer participants in the SWIS condition exceeded the cut-off score at post-test (− 11%). The WLC condition shows the opposite effect with an increase of participants with clinically relevant depressive symptoms (+ 8%). This result presents a superior effect of the SWIS condition regarding the development of clinically relevant depressive symptoms.

### Long-term effects of SWIS

The long-term effects of SWIS are displayed in Table 4. Participants of the WLC were included in this analysis if they did not differ from the SWIS condition at T1. Nearly all dependent variables met this requirement, except for maladaptive stress coping strategies. Furthermore, only participants who completed all measurements from T1 to T3 of the WLC were included (*N* = 5). Using these criteria, data from five additional WLC participants were added in the remaining eight dependent variables. Again, the scales panic and alcohol abuse were excluded from the analysis due to the fact that most students did not report any impairment.

**Table 4:** Mental health long-term effects of SWIS

Variable	N	Pre-test _1_	Post-test _2_	Follow-up _3_	Test statistic	p	Post-hoc	p_adj_	Effect size d / r
		M (SD)	M (SD)	M (SD)	F / Z				
Mental health									
Depression	19	21.47 (9.71)	20.32 (9.36)	17.47 (10.88)	F(2,17) = 1.212	.322	–	–	–
Somatic complaints	18	7.72 (4.44)	6.44 (3.96)	6.44 (4.02)	***F(2,16) = 3.509***	***.055***	***t***_***1–2***_ ***= 2.336***	***.096***	***d = .55***
							***t***_***1–3***_ ***= 2.426***	***.082***	***d = .57***
							t_2–3_ = n.s.	–	–
Anxiety	11	8.09 (2.02)	7.36 (2.11)	6.36 (2.25)	**F(2,9) = 1.832**	**.015**	t_1–2_ = n.s.	–	–
							**t**_**1–3**_ **= 3.846**	**.010**	**d = 1.16**
							***t***_***2–3***_ ***= 2.801***	***.056***	***d = .84***
General stress	18	7.11 (3.58)	6.56 (3.89)	5.77 (2.94)	F(2,16) = 1.832	.192	–	–	–
Stress coping strategies									
Adaptive strategies	19	17.92 (2.42)	16.08 (4.01)	15.70 (5.02)	Z(2,19) = 1.425	.491	–	–	–
Maladaptive strategies	15	19.08 (3.47)	18.46 (4.23)	16.48 (5.20)	Z(2,15) = 3.138	.208	–	–	–
Quality of life									
Physical condition	19	2.97 (.73)	3.42 (.58)	3.57 (.70)	**F(2,17) = 7.401**	**.005**	**t**_**1–2**_ **= −3.000**	**.023**	**d = −.69**
							**t**_**1–3**_ **= −3.903**	**.003**	**d = −.89**
							t_2–3_ = n.s.	–	–
Cognitive emotional condition	19	2.94 (.59)	3.01 (.53)	3.22 (.57)	F(2,17) = 2.555	.107	–	–	–
Quality of life	19	3.25 (.61)	3.39 (.54)	3.59 (.50)	**F(2,17) = 3.670**	**.047**	t_1–2_ = n.s.	–	–
							**t**_**1–3**_ **= −2.656**	**.047**	**d = −.61**
							t_2–3_ = n.s.	–	–

Bold characters indicate significant results (*p* < .05). Bold italics indicate tendentially significant results (*p* < .10). – indicate statistically insignificant results. All post-hoc tests were adjusted for alpha error accumulation

Three months after participation at the SWIS-training, the students reported fewer somatic complaints, less anxiety, an improved physical condition as well as a better quality of life with medium to large effect sizes. The significant improvements were mainly observed between pre- and follow-up measurement, indicating an improvement after the conclusion of the training. The other investigated outcome measures showed descriptive improvements, including less depressive symptoms, less stress, fewer maladaptive stress coping strategies and an improved cognitive emotional condition. The reduction of depressive symptoms remained stable under the clinical cut-off score of the ADS.

#### Depressive symptoms and long-term mental health development

Participants with and without clinically relevant depressive symptoms at pre-test (ADS < 23) were compared regarding their long-term mental health development. Descriptive statistics are displayed in Appendix 1. Group (depressive vs. non-depressive), time (pre- to follow-up measurement) and group x time interactions are presented in Appendix 2.

There were several group and time effects, but few group x time interaction effects. The general stress declined more rapidly in participants with clinically relevant depressive symptoms. Aside from that interaction, there were no further significant interaction effects. Hence, the presence or absence of clinically relevant depressive symptoms did not affect the development of the long-term mental health outcomes.

#### Clinically significant changes

**Fig. 3:**
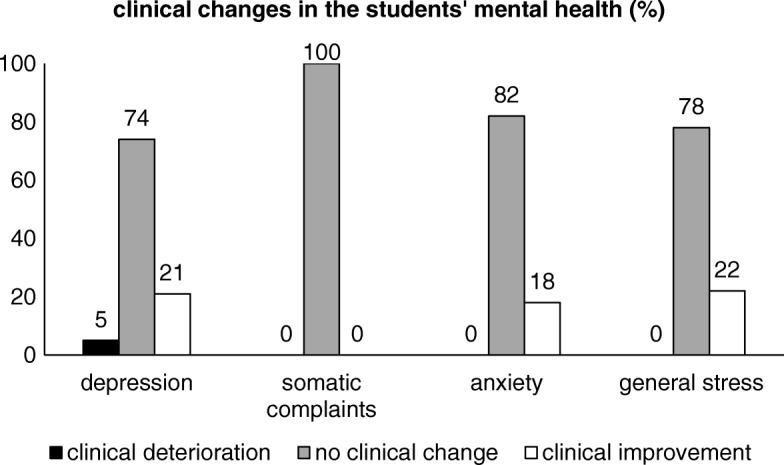
Long-term clinical changes in the students’ mental health

The interpretation of the RCIs are provided in Appendix 3. Figure 3 provides an overview of the long-term clinical changes in the mental health variables. Approximately one fifth of the students reported clinically improved depression, anxiety and general stress. The students’ somatic complaints remained stable. Only one student (5%) showed deteriorated depression scores in the long-term.

**Fig. 4:**
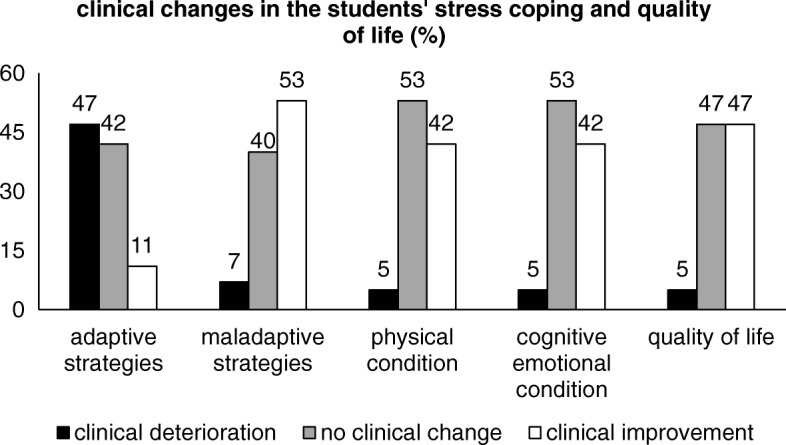
Long-term clinical change in the students’ stress coping strategies and quality of life

Figure 4 displays the long-term clinical changes of the students’ stress coping strategies and quality of life. The students’ reported divergent results regarding their stress coping strategies: While 47% showed a clinically relevant deterioration of their adaptive strategies, 53% reported a clinically relevant improvement of their maladaptive stress coping strategies.

Forty two percent of the students showed a clinically improved physical and cognitive emotional condition in the long-term. Regarding their general quality of life, nearly half of the sample (47%) reported clinical improvements. Again, only one student (5%) showed a clinically relevant reduction of the physical, cognitive emotional and general quality of life.

The analyses of the long-term clinical changes regarding the students’ mental health, stress coping strategies and quality of life indicate moderate improvements on all scales, with the exception of the adaptive stress coping strategies.

### Completer analyses

Fifteen of 56 participants were lost to attrition from pre- to post-measurement (− 27%). Of those 15 participants, 7 participants did not complete the SWIS training (− 13%) and 8 participants completed the SWIS training, but did not complete the post-measurement (− 14%). The completers did not differ from the non-completers on any dependent variable, age, or gender (all *p* > .05). Twenty two of 41 participants were lost to attrition from post- to follow-up measurement (− 54%). Again, the completers did not differ from the non-completers on any dependent variable, age, or gender (all *p* > .05).

## Discussion

This randomized controlled pilot trial investigated the effects of SWIS compared with WLC concerning mental health, stress coping strategies and quality of life in university students, as well as long-term effects.

The depression scores of the students declined significantly in both conditions. Additionally, participants in the SWIS condition reported descriptively less anxiety and general stress than the WLC. Except for a significant increase in adaptive stress coping strategies in WLC students, none of the stress coping strategies changed significantly. Beyond, students had a significantly improved physical condition after the SWIS training, which was not observed in the WLC condition. However, the other two scales (cognitive emotional condition and quality of life) did not change significantly. From a clinical perspective, the SWIS condition was superior compared to the WLC condition regarding the reduction of clinically relevant depressive symptoms.

In the long-term, the students in the SWIS condition reported statistically significant fewer somatic complaints, less anxiety, an improved physical condition and a better quality of life from pre-test to follow-up with moderate to large effects. Depressive symptoms only had a statistically significant impact on general stress. Furthermore, moderate long-term clinically relevant changes occurred in most mental health variables and in the students’ quality of life.

Completer analyses did not reveal any significant differences between completers and non-completers at post- or follow-up measurement. Overall, the SWIS sleep training differed only slightly from the WLC condition. However, positive long-term results support the additional benefits of SWIS on the mental health and quality of life in college and university students.

### Interpretation of results

The discrepancies between the insignificant results directly after the training and the various significant long-term effects indicate that the students need more time to implement the strategies they obtained in the SWIS training. Depressive symptoms did not affect the positive long-term development of the students’ mental health in nearly all variables but general stress. The moderate clinical long-term improvements concurred with the statistical analyses. This presents SWIS as a feasible and effective program for students who suffer from sleep problems as well as depressive symptoms.

These improvements were expected, as SWIS specifically targets the investigated variables. For example, sleep hygiene instructions also include rules about healthy eating, alcohol consumption, and physical activity. These aspects might reduce somatic complaints and consequently improve the students’ physical condition (e.g. less stomach aches, less skin problems). Furthermore, improved sleep is connected directly with an improved physical condition. Anxiety issues are tackled with cognitive restructuring and relaxation techniques, such as progressive muscle relaxation or hypnotherapeutic elements. Finally, the stress coping strategies and the general self-care character of SWIS help to improve the students’ quality of life.

The most obvious conflict regarding these results was the statistically significant improvement of the depression scores and adaptive stress coping strategies in the WLC condition. This may be explained by several artefacts including statistical regression to the mean, effects of the diagnostic sleep log and unspecific expectation effects in WLC conditions that were reported in previous publications [30]. Furthermore, the detailed analysis of ADS cut-off score revealed that more WLC participants reported clinically relevant depressive symptoms at post-test in comparison with the pre-test percentages and with the SWIS condition. Therefore, the statistically significant improvements of the WLC participants’ depressive symptoms were not observed on a clinical level.

The completer analyses revealed that there were no differences between completers and non-completers at post- and follow-up measurement. This result shows that the study itself did not keep specific groups from participating, leading to the reverse conclusion that the students accepted SWIS regardless of their baseline characteristics.

Adverse effects were only inferred by a descriptive decline of the adaptive stress coping strategies in the SWIS condition. However, this reduction was not significant, rendering its interpretation negligible.

### Scientific integration

#### Mental health

A recent review [10] provided evidence that sleep trainings improve the mental health of university students, although differences regarding the type of intervention emerged. While studies on sleep hygiene, CBT and other psychotherapeutic approaches reported small to medium effect sizes for mental health outcomes, the studies with relaxation trainings had large effect sizes. As SWIS combines CBT-I and HT-I, it also includes relaxation elements (e.g. PMR). However, comparison to other studies is impaired by a lack of mental health outcome reporting and insignificant results. Only 37% of the investigated sleep trainings showed significant improvements in mental health variables, 11% did not find significant changes and 52% did not report any mental health variables at all. Despite the small sample size, the current study revealed similar effect sizes.

In detail, two similar sleep interventions employed CBT to improve sleep in college students [31], and in students with insomnia according to DSM-5 [32]. Morris and colleagues randomized 95 healthy students into an online CBT condition (60% women) or a WLC (70% women) [31]. After the six weekly intervention sessions the students reported significantly less depression in both groups. However, the authors did not find any interaction effect. This corresponds with our results, as depression scores decreased in both conditions (SWIS and WLC) immediately after the training. The other CBT study by Taylor and colleagues investigated several mental health outcomes in 34 college students, with 17 students in each condition (59% women; CBT vs. WLC) [32]. Compared to the students in the WLC condition, those in the CBT condition reported significantly less general fatigue. Other investigated mental health aspects like quality of life, anxiety, depression, stress, alcohol and marijuana use did not change significantly after the sleep training [32]. This indicates that the SWIS training as a CBT-I and HT-I combination has a larger positive effect on the students’ mental health and quality of life than similar programs that focus solely on CBT.

#### Stress coping

The students’ stress coping strategies were not influenced by the SWIS sleep training. This conflicts with the findings of Pillai and colleagues, who reported that the relationship between stress and sleep is mediated by the three stress coping strategies behavioral disengagement, distraction and substance abuse [7]. However, these three stress coping behaviors only cover a small proportion of the stress coping behaviors that were examined in the present study. While behavioral disengagement and substance abuse can be assigned to the ten maladaptive strategies measured by the stress coping inventory, distraction belongs to the seven adaptive strategies. Thus, a more detailed examination of the different stress coping strategies, especially behavioral disengagement, substance abuse and distraction, might reveal further differences and should be analyzed in further studies.

#### Long-term effects

The investigation of long-term effects in the current study revealed significant improvements of the students’ somatic complaints, anxiety, physical condition and quality of life. The follow-up data of Taylor and colleagues [32] did not present these mental health effects. However, the authors observed a significant improvement from post- to follow-up measurement regarding several sleep-related variables (e.g. dysfunctional beliefs, daytime impairments and general fatigue), which indicates incubation effects [32]. These effects may also be present in the current study, e.g. because the students need more time to implement the strategies that they learned in the SWIS sleep training. Beyond, studies regarding adolescents and children also reported a further enhancement 3 months after treatment [16, 18].

#### Completer analyses

Attrition was 27% at post-measurement and 54% at follow-up measurement. The larger attrition rate for the third assessment was not surprising, as the follow-up measurement was taken online, thus reducing the participants’ feelings of obligation to comply with the procedure. This assumption is supported by similar results in the CBT-I study conducted by Taylor and colleagues [32]: There, 15% of the students did not complete the post-measurement and 56% were lost to attrition at 3 months follow-up measurement. Furthermore, a recent meta-analysis on drop-out rates in CBT-studies suggests that studies with depressed participants and outpatients had higher drop-out rates than studies with non-depressed inpatient participants [33]. As the current study contained 29 participants (56%) with clinically relevant depressive symptoms and an outpatient setting, higher drop-out rates can be expected.

### Strengths and limitations

The randomized controlled design and the 3 month follow-up are important strengths of the current study. The comparison with other sleep trainings revealed the importance of long-term measurements, as the students have to implement learned strategies in daily routine. Although the general dropout rates were high, most of the students that started the training completed it (SWIS: 82%, WLC: 85%), supporting the feasibility and acceptance of SWIS. Furthermore, the investigated university students all suffered from sleep disorders or had an impaired sleep quality. The inclusion of participants with subclinical sleep impairments might result in a reduced efficacy of the SWIS sleep training due to the fact that the participants did not have severe sleep problems to begin with, creating a ceiling effect. Therefore, further studies should compare healthy participants to subclinical and clinical participants to examine sleep-related ceiling effects. Finally, the examination of statistically as well as clinically significant changes provides a better understanding of the students’ long-term development.

One limitation of the current study was the small and unequal sample size. Despite the fact that the sample size exceeded the necessary 33 participants, the unequal sample sizes in the different conditions impaired statistical calculations. Another limitation is the WLC design. Recent reviews showed that WLCs may inflate intervention effect sizes [34]. Correspondingly, a review of meta-analysis found that CBT is effective compared to waiting list, but mixed results occur when comparing CBT to active control conditions or other interventions [35]. A meta-analysis investigating the effects of waiting list conditions in depression even resulted in nocebo effects, as waiting list conditions were less effective than no treatment conditions [36]. Consequently, future studies should include other control conditions. Furthermore, a long-term investigation of the WLC would have been the best design. However, such a design was not possible due to the fact that the students in the WLC received the SWIS training directly after they completed their second assessment. Due to a lack of information concerning the instruments’ stabilities, real life changes in symptoms might also be attributed to a low test retest reliability. Finally, the instrument that used filter questions to investigate panic symptoms and alcohol abuse was not appropriate for such a small sample size, as only a few students confirmed that they ever had any of the aforementioned symptoms. This resulted in missing data for most of the panic and alcohol scales.

## Conclusions

In the long-term, the SWIS sleep training improved not only sleep, but also different aspects of university students’ mental health and quality of life. Stress coping strategies did not change significantly, although a more detailed analysis might be necessary. The positive developments between post-test and follow up in the current study again showed the importance of long-term measurements. The presence of absence of depressive symptoms at pre-measurement did not impact the long-term development of the students’ mental health, leading to the conclusion that the SWIS sleep training is similarly effective for depressed and non-depressed students.

Future studies should include a larger sample size. This would allow a subdivision into impaired and non-impaired students, which facilitates the detection and avoidance of ceiling effects. Another advantage of a larger sample size would be the possibility to apply the appropriate statistical tests (e.g. repeated measures ANOVA). Furthermore, future studies should include different control conditions, e.g. alternative interventions, active control conditions, and treatment as usual. Finally, future studies should also consider alternative instruments for the measurement of panic symptoms and alcohol abuse and find ways to reduce attrition rates, e.g. by reimbursing the participants.

## Appendix 1

**Table 5:** Means and standard deviations of the long-term effects in participants with clinically relevant depressive symptoms and non-clinically relevant depressive symptoms

Variable		N	Pre-test	Post-test	Follow-up
			M (SD)	M (SD)	M (SD)
Mental health					
Depression	ADS < 23	10	13.80 (5.39)	15.70 (8.59)	12.70 (6.24)
	ADS ≥23	9	30.00 (4.89)	25.44 (7.60)	22.78 (12.75)
Somatic complaints	ADS < 23	10	8.30 (3.83)	6.70 (3.89)	6.80 (4.63)
	ADS ≥23	8	7.00 (5.29)	6.12 (4.29)	6.00 (3.34)
Anxiety	ADS < 23	6	7.83 (1.94)	7.33 (2.16)	6.33 (1.97)
	ADS ≥23	5	8.40 (2.30)	7.40 (2.30)	6.40 (2.79)
General stress	ADS < 23	10	4.80 (2.86)	5.00 (3.89)	5.10 (3.14)
	ADS ≥23	8	10.00 (3.58)	8.50 (3.02)	6.63 (2.62)
Stress coping strategies					
Adaptive strategies	ADS < 23	10	17.90 (2.43)	15.86 (3.96)	16.32 (4.72)
	ADS ≥23	9	17.95 (2.55)	16.32 (4.29)	15.01 (5.54)
Maladaptive strategies	ADS < 23	8	17.13 (3.17)	16.47 (4.77)	16.56 (4.77)
	ADS ≥23	7	21.32 (2.31)	20.75 (1.95)	16.39 (6.04)
Quality of life					
Physical condition	ADS < 23	10	3.20 (.61)	3.55 (.72)	3.62 (.82)
	ADS ≥23	9	2.70 (.80)	3.27 (.35)	3.51 (.59)
Cognitive emotional condition	ADS < 23	10	3.32 (.45)	3.23 (.54)	3.43 (.57)
	ADS ≥23	9	2.52 (.41)	2.77 (.42)	2.99 (.49)
Quality of life	ADS < 23	10	3.67 (.39)	3.63 (.57)	3.76 (.53)
	ADS ≥23	9	2.79 (.44)	3.14 (.39)	3.41 (.43)

## Appendix 2

**Table 6:** Long-term effects of SWIS in depressed and non-depressed participants: Group, time and group x time interaction effects

Variable	Group (depression)	Time	Group x time interaction
Mental health			
Depression	F(1,19) = 20.638, *p* = .000	–	–
Somatic complaints	–	F(2,15) = 3.157, *p* = .072	–
Anxiety	–	F(2,8) = 6.377, *p* = .022	–
General stress	F (1,18) = 7.291, *p* = .016	F(2,15) = 4.121, *p* = .037	F(2,15) = 6.142, *p* = .011
Stress coping strategies			
Adaptive strategies	–	–	–
Maladaptive strategies	–	F(2,12) = 2.898, *p* = .094	–
Quality of life			
Physical condition	–	F(2,16) = 7.862, *p* = .004	–
Cognitive emotional condition	F(1,19) = 10.146, *p* = .005	–	–
Quality of life	F(1,19) = 11.200, *p* = .004	F(2,16) = 4.666, *p* = .025	–

– indicate statistically insignificant results

## Appendix 3

### Interpretation of the RCIs

ADS depressive symptoms ➔ RCI = 9 points

≥ + 9 points = **improvement**

≤ − 9 points = **deterioration**

-9 to + 9 points = no clinical change

PHQ somatic complaints ➔ RCI = 6 points

≥ + 6 points = **improvement**

≤ − 6 points = **deterioration**

-6 to + 6 points = no clinical change

PHQ anxiety ➔ RCI = 4 points

≥ + 4 points = **improvement**

≤ − 4 points = **deterioration**

-4 to + 4 points = no clinical change

PHQ general stress ➔ RCI = 5 points

≥ + 5 points = **improvement**

≤ − 5 points = **deterioration**

-5 to + 5 points = no clinical change

SVF adaptive strategies ➔ RCI = 2.21

≥ + 2.21 points = **deterioration**, because this would indicate that the pre-test score was higher than the follow-up score, which would represent a decline in adaptive stress coping strategies

≤ − 2.21 points = **improvement**

-2.21 to + 2.21 points = no clinical change

SVF maladaptive strategies ➔ RCI = 2.16

≥ + 2.16 points = **improvement**

≤ − 2.16 points = **deterioration**

-2.16 to + 2.16 points = no clinical change

SEL physical condition ➔ RCI = .69

≥ + .69 points = **deterioration**, because higher scores indicate a better physical condition. Therefore, a positive change score (pre – follow-up) would mean that the participants had a better physical condition at pre-test compared to the follow-up measurement.

≤ − .69 points = **improvement**

-.69 to +.69 points = no clinical change

SEL cognitive-emotional condition ➔ RCI = .47

≥ + .47 points = **deterioration**

≤ − .47 points = **improvement**

-.47 to +.47 points = no clinical change

SEL quality of life ➔ RCI = .41

≥ + .41 points = **deterioration**

≤ − .41 points = **improvement**

-.41 to +.41 points = no clinical change

## Abbreviations

ADS: Allgemeine Depressionsskala (general depression scale)
CBT-I: Cognitive behavior therapy for insomnia
DSM: Diagnostic and Statistical Manual of Mental Disorders
HT-I: Hypnotherapy for insomnia
PHQ-D: Patient Health Questionnaire (PHQ-D = German version)
PMR: Progressive muscle relaxation
RCI: Reliable change index
SEL: Skalen zur Erfassung der Lebensqualität (quality of life scales)
SVF-78: Stressverarbeitungsfragebogen (Stress Coping Questionnaire)
SWIS: Studieren wie im Schlaf (StudySleep)
WLC: Waiting list control condition

## Footnotes

1 d = $$ \frac{t}{\sqrt{N}} $$

2 r = $$ \frac{z}{\sqrt{N}} $$
